# ICU-Onset *Clostridium difficile* Infection in a University Hospital in China: A Prospective Cohort Study

**DOI:** 10.1371/journal.pone.0111735

**Published:** 2014-11-05

**Authors:** Xiaohui Wang, Lin Cai, Rujia Yu, Wenzhi Huang, Zhiyong Zong

**Affiliations:** 1 Center of Infectious Diseases, West China Hospital, Sichuan University, Chengdu, China; 2 Division of Infectious Diseases, State Key Laboratory of Biotherapy, Chengdu, China; 3 Department of Intensive Care Unit, West China Hospital, Sichuan University, Chengdu, China; 4 Department of Infection Control, West China Hospital, Sichuan University, Chengdu, China; University of Ulster, United Kingdom

## Abstract

A prospective study was conducted to investigate the incidence, clinical profiles and outcome of ICU-onset CDI in a 50-bed medical ICU at a university hospital in China. Stools were collected from patients who developed ICU-onset diarrhea and was screened for *tcdA* (toxin A gene) and *tcdB* (toxin B gene) by PCR. CDI cases were compared with the ICU-onset non-CDI diarrhea cases for demographics, comorbidities, potential risk factors, major laboratory findings and outcomes. Stool samples from CDI cases were subjected to *C. difficile* culture and *C. difficile* isolates were screened for *tcdA*, *tcdB* and the binary toxin genes (*cdtA* and *cdtB*) using multiplex PCR. Strain typing of toxigenic *C. difficile* isolates was performed using multilocus sequence typing. There were 1,277 patients in the ICU during the study period and 124 (9.7%) developed ICU-onset diarrhea, of which 31 patients had CDI. The incidence of ICU-onset CDI was 25.2 cases per 10,000 ICU days. ICU-onset CDI cases had similar features with ICU-onset non-CDI diarrhea cases including the use of proton pump inhibitors and antibacterial agents. The crude mortality rate of ICU-onset CDI was 22.6%, but the attributable mortality rate of ICU-onset CDI was only 3.2% here. Toxigenic *C. difficile* isolates were recovered from 28 out of the 31 patients with CDI. *cdtA* and *cdtB* were found in two strains. Seventeen STs including 11 new STs were identified. All of the 11 new STs were single-locus variants of known STs and the 17 STs identified here could be clustered into 3 clades. The incidence of ICU-onset CDI here is similar to those in Europe and North America, suggesting that CDI is likely to be a common problem in China. Toxigenic *C. difficile* here belonged to a variety of STs, which may represent a significant clonal expansion rather than the true clonal diversity.

## Introduction

*Clostridium difficile*, a Gram-positive spore-forming anaerobic bacterium, is the leading cause of nosocomial diarrhea in industrialized countries [1], [2]. Clinical manifestations of *C. difficile* infection (CDI) ranged from mild diarrhea to pseudomembranous colitis and the use of antibacterial agents is the major risk factor for the development of CDI [3]. Although CDI has been well recognized in Europe and North America [1], [4], little is known about the prevalence of CDI and the clonal relatedness of *C. difficile* isolates in China [5]. This may be due to the lack of awareness and the absence of laboratory capacity to detect this nosocomial pathogen in most hospitals in China.

Patients in intensive care units (ICU) commonly received intensified antibacterial therapy and may therefore develop CDI. Furthermore, ICU patients may have a poor outcome when they developed CDI as they usually had comorbidities [6]. Surprisingly, studies on the incidence and outcome of CDI cases in ICU are scarce [6]. Available studies are typically retrospective and therefore might underestimate the incidence.

## Material and Methods

### Prospective cohort study

A prospective study was conducted to investigate the incidence, clinical profiles and outcome of ICU-onset CDI and to determine the clonal relatedness of ICU-onset toxigenic *C. difficile* isolates in a 50-bed medical ICU at West China Hospital, Sichuan University, Chengdu, southwest China, during a period of 35 weeks between May 2012 and January 2013.

Stools were collected from patients who developed ICU-onset diarrhea, which was defined as three or more loose stools per day for at least one day occurring within their stay in ICU. Diarrhea occurred during the stay in hospital but in an unit other than ICU was not considered as ICU-onset. The diarrhea in all of these patients occurred later than 48 hours after admission to the hospital and therefore were considered nosocomial. Total DNA of stool samples was prepared using the Stool DNA Kit (OMEGA, Norcross, GA) and was screened for the pathogenicity locus operon (Paloc) genes, *tcdA* (toxin A gene) and *tcdB* (toxin B gene) by PCR as described previously [7]. Of note, PCR for *tcdA* could yield 369-bp or 110-bp amplicons and samples with 110-bp amplicons were defined as negative for *tcdA* [7]. Patients with diarrhea who had a stool sample tested positive to either *tcdA* or *tcdB* were CDI cases.

Patients with ICU-onset diarrhea were tracked during the whole period of their stay in the hospital by the study team. The medical records of these patients were retrieved to compare CDI cases with the remaining ICU-onset non-CDI diarrhea cases from which stool DNA was negative to both *tcdA* and *tcdB* for demographics, comorbidities, potential risk factors, major laboratory findings and outcomes (Table 1). Whether CDI was an attributable cause, a contributing cause, or unrelated to the cause of death was judged by two physicians independently for each death based on the conclusion whether the patients would die or not if they did not have CDI. In the case of a disagreement, a consensus was reached after discussion.

**Table 1:** Features and outcomes of ICU-onset CDI and non-CDI diarrhea.

Feature^a^	ICU-onset CDI (n = 31)	ICU-onset non-CDI diarrhea (n = 93)	*P* value
Age (years-old), mean±IQR	59.00±35.00	69.00±30.00	0.204
No. of cases ≥ 65-years-old	13 (41.9%)	39 (41.4%)	1.000
Sex, male/female	25/6	57/36	0.049
Diabetes	2 (6.5%)	20 (21.5%)	0.057
Malignancy	5 (16.1%)	15 (16.1%)	1.000
COPD	4 (12.9%)	15 (16.1%)	0.886
Pancreatitis	1 (3.2%)	13 (13.9%)	0.190
Renal failure	2 (6.5%)	20 (21.5%)	0.057
Liver failure	6 (19.3%)	8 (8.6%)	0.190
Nasogastric tube	30 (96.8%)	80 (86.0%)	0.190
APACHE II score on ICU admission median±IQR	21.00±14.00	23.00±12.50	0.072
SOFA score on ICU admission median±IQR	10.00±4.00	11.00±4.00	0.132
Charlson comorbidity index, median±IQR	4.00±5.00	5.00±4.00	0.429
Use of antibacterials^b^ (%)	31 (100%)	90 (96.8%)	0.572
β-lactams/β-lactamase inhibitors	21 (67.74%)	78 (83.87%)	0.053
Carbapenems	14 (45.16%)	40 (43.01%)	0.834
1st cephalosporins	2 (6.45%)	1 (1.08%)	0.154
2nd-generation cephalosporins	0	2 (2.15%)	1.000
3rd-generation cephalosporins	3 (9.68%)	6 (6.45%)	0.842
4th-generation cephalosporins	1 (3.23%)	0	0.250
Aztreonam	3 (9.68%)	0	0.014
Cephamycins	2 (6.45%)	5 (5.38%)	1.000
Fluoroquinones	7 (22.58%)	21(22.58%)	1.000
Vancomycin	3 (9.68%)	14 (15.05%)	0.651
Clindamycin	0	1 (1.08%)	1.000
SMZ-TMP	0	1 (1.08%)	1.000
Tetracyclines	1 (3.23%)	2 (2.15%)	1.000
Linezolid	1 (3.23%)	2 (2.15%)	1.000
No. of antibacterials^b^, median (range)	2 (1–5)	2 (0–7)	
Receiving chemotherapy (%)	4 (12.90%)	9 (9.68%)	0.866
Receiving any GAS (%)	28 (90.32)	85 (91.40%)	1.000
PPI	28 (90.32%)	83 (89.25%)	1.000
Histamine-2 receptor antagonists	0	2 (2.15%)	1.000
Maximum WBC c, median±IQR	10.08±8.76	10.27±8.49	0.390
Maximum Temperature (°C) c, median±IQR	38.00±1.60	38.10±1.55	0.970
Maximum creatine c, median±IQR	63.00±74.00	124.00±184.40	0.000
LOS in hospital (days), median±IQR	28.00±35.00	33.00±35.50	0.956
LOS in ICU (days), mean±SD	31.74±43.21	23.25±20.06	0.298
LOS before onset (days), median±IQR	11.00±12.00	8.00±14.00	0.068
Death in hospital (mortality rate)	7 (22.6%)	(28%)	0.557

a IQR, interquartile range, for data that did not fit a normal distribution; SD, standard deviation, for data with a normal distribution.

b Used within 1 month prior to onset of diarrhea. None of patients with CDI or non-CDI diarrhea received aminoglycosides or macrolides.

c Collected on the day of onset or two days before.

### Culture and strain typing

Stool samples from CDI cases that were positive to either *tcdA* or *tcdB* by PCR were treated with absolute ethanol, streaked onto cefoxitin cycloserine fructose agar (CCFA; OXOID, Basingstoke, UK) plates and incubated at 37°C for 72 hours. Colonies with the characteristic odor were subjected to Gram stain and multiplex PCR for detecting the presence of *tpi* (encoding triose phosphate isomerase of *C. difficile*), *tcdA*, *tcdB* and the binary toxin genes, *cdtA* and *cdtB*, as described previously [7], [8]. *C. difficile* strains that were positive to either *tcdA* or *tcdB* were toxigenic strains.

Multilocus sequence typing (MLST) was performed for the toxigenic *C. difficile* strains as described previously [9]. For the three CDI patients where no toxigenic *C. difficile* was recovered from stool, total stool DNA was directly used for *C. difficile* MLST [9]. eBURST (version 3, http://eburst.mlst.net/) was used to assign sequence types (STs) to clonal complexes (CCs), which were defined as those sharing identical alleles at six of seven loci and were named after the predicted founder ST. STs of *C. difficile* were also clustered into clades by phylogenetic analysis using the concatenated sequences of the seven loci of MLST as described previously [9].

### Statistical analysis

Statistical analysis was performed using SPSS version 18.0 (SPSS Inc., Chicago, IL). Differences in proportions were compared using a chi-squared test, while differences in median values were examined using a Mann-Whitney U test. The difference in LOS in ICU, which is in normal distribution, was examined using a Student's *t*-test.

### Ethics statement

This study was approved by the Ethics Committee of West China Hospital and the need of patient consents was waived by the Ethics Committee.

## Results, Discussion, and Conclusions

During the study period, there were 1,277 patients in the ICU and 124 (9.7%) developed ICU-onset diarrhea, of which 24 had a stool sample that tested positive for *tcdA* and *tcdB* (A+B+) and 7 had a sample positive for *tcdB* only (A-B+). Therefore, a total of 31 patients had CDI, accounting for 2.4% of the ICU patient population and 25.0% of those with ICU-onset diarrhea. The incidence of ICU-onset CDI was 25.2 cases per 10,000 ICU days, which is close to the 32 cases per 10,000 ICU days found in a medical ICU in the USA [10]. The ICU-onset CDI rate (2.4%) in this study is slightly lower than that in two UK ICUs (one medical-surgical and one trauma) [11] (3.3%, 62/185) but is significantly higher than rates found in multiple medical or medical-surgical ICUs in France (0.9%, 47/5260) [12] and in six medical-surgical ICUs in Canada (1.5%, 236/15314) [13]. Variations in the incidence and rate of ICU-onset CDI in different studies may be due to the type and location of ICUs and local infection control practices.

To identify risk factors for developing CDI and to demonstrate whether CDI was associated with poorer outcomes, CDI cases had commonly been compared with non-CDI cases in a few previous studies, e.g. [14]–[16]. However, most non-CDI cases had no diarrhea. Therefore, risk factors and outcome features identified for CDI through such comparisons are unlikely specific for CDI but may simply reflect for diarrhea as a whole. To overcome the shortcoming, for the 124 patients with ICU-onset diarrhea in this study, CDI cases (n = 31) were compared with the remaining 93 ICU-onset non-CDI diarrhea cases with characteristics listed in Table 1. Among 31 ICU-onset CDI cases, 13 (41.9%) were 65-year-old or above, 20 (64.5%) had at least one comorbidity and all received at least one antibacterial agent, most (28/31, 90.3%) of which were carbapenems or compounds containing β-lactamase inhibitors, within one month prior to the onset of CDI (Table 1). The interval between admission to ICU and onset of CDI ranged from 3 to 72 days (median 11 days). Overall, ICU-onset CDI cases features were similar to ICU-onset non-CDI diarrhea cases, except that non-CDI cases were associated with higher creatine levels and patients receiving aztreonam were more likely to develop CDI (Table 1). The differences between CDI and non-CDI diarrhea cases warrant further investigation. Of note, the use of proton pump inhibitors (PPIs) has been identified as a risk factor for developing CDI by comparing CDI and non-CDI cases in a few reports, e.g. [16]. However, here most cases with non-CDI nosocomial diarrhea also received PPIs prior to the onset of diarrhea (Table 1) and therefore the use of PPIs might not be a risk factor specific for the acquisition of CDI but rather a risk factor for developing nosocomial diarrhea. Similarly, age>65 years, using antibacterial agents, receiving chemotherapy and an indwelling nasogastric tube, which have all been well recognized as risk factors for developing CDI [14]–[16], might actually be risk factors for developing nosocomial diarrhea regardless of the etiology.

No patients with ICU-onset CDI needed renal dialysis or underwent colectomy but seven such patients died during their stay in hospital, corresponding to a crude mortality rate of 22.6%, which is consistent with crude mortality rates of ICU-onset CDI of 21.5% to 36.7% in several studies in Europe and North America [12], [17]. Previous studies have not demonstrated the association of CDI with a higher hospital mortality rate [12], [17]. However, here the crude mortality rate of ICU-onset CDI cases was higher than the overall mortality rate (16.4%) of all ICU patients during the same period but was similar to that (28.0%) of ICU-onset non-CDI diarrhea cases. This suggests that nosocomial diarrhea as a whole, rather than CDI specifically, was associated with a higher mortality rate in ICU.

Among the seven patients who died, CDI was considered the attributable cause for one and a contributing cause for another two. Correspondingly, the attributable mortality rate of ICU-onset CDI was 3.2% (1/31) here. Previous studies have demonstrated that attributable mortality rates of CDI ranged from 5.5% to 6.9% for all patients (including non-ICU patients) but reach as high as 16.7% in outbreaks [6]. The reason for a lower attributable mortality rate here remains unclear but may be due to the lack of an objective method to determine the attributable cause of death or the fact that the number of cases may be too low here.

Toxigenic *C. difficile* isolates were recovered from 28 out of the 31 patients with CDI, including 22 A+B+ and 6 A−B+ strains (Table 2). Of note, A−B+ strains are relatively common in China, accounting for 17–34% of all toxigenic strains [18], [19]. *cdtA* and *cdtB* were found in only two A+B+ strains (2/31, 6.5%). Binary toxin genes have been detected in 23% of toxigenic *C. difficile* in Europe [4]. In contrast, strains carrying binary toxin genes remain very rare in China [18] with two found in this study.

**Table 2:** The STs and toxin gene carriage of *C. difficile* strains.

ST^a^	Allele profile^b^	Ribotype^c^	CC	Clade^d^	Toxin A	Toxin B	Binary toxin	No. of strains
ST2	1-1-2-1-5-3-1	014/020	28	1/HA1	+	+	−	1
ST3	1-1-2-1-1-1-1	001	28	1/HA1	+	+	−	5
ST8	1-1-2-6-1-5-1	002	28	1/HA1	+	+	−	1
ST35	2-5-8-1-1-3-6	046	28	1/HA1	+	+	−	4
ST37	3-7-3-8-6-9-11	017	37	4/A-B+	−	+	−	3
ST54	1-4-7-1-1-3-3	012	28	1/HA1	+	+	−	4
ST208	2-7-8-1-1-3-6		28	1/HA1	+	+	−	1
ST209	1-6-4-7-2-8-11		5	3/HA2	+	+	+	2
ST210	1-1-2-1-1-1-11		28	1/HA1	+	+	−	1
ST211	2-5-8-1-1-3-11		28	1/HA1	+	+	−	1
ST212	1-4-7-1-1-3-6		28	1/HA1	+	+	−	1
ST213	1-11-6-6-1-12-6		99	1/HA1	+	+	−	1
ST214	1-1-2-1-5-3-12		28	1/HA1	+	+	−	1
ST215	3-7-3-8-6-9-29		37	4/A−B+	−	+	−	1
ST216	3-7-3-8-6-9-6		37	4/A−B+	−	+	−	1
ST218	2-5-8-1-1-3-1		28	1/HA1	+	+	−	1
ST219	3-7-3-1-6-9-11		37	4/A-B+	−	+	−	2

a Underlined STs include results from directly typing the stool DNA from three CDI patients with no recovery of *C. difficile*.

b Alleles are *adk-atpA-dxr-glyA-recA-sodA-tpi.*

c Ribotype is predicted and those for new STs are not available.

d Names of clades are from reference [9].

**Figure 1:**
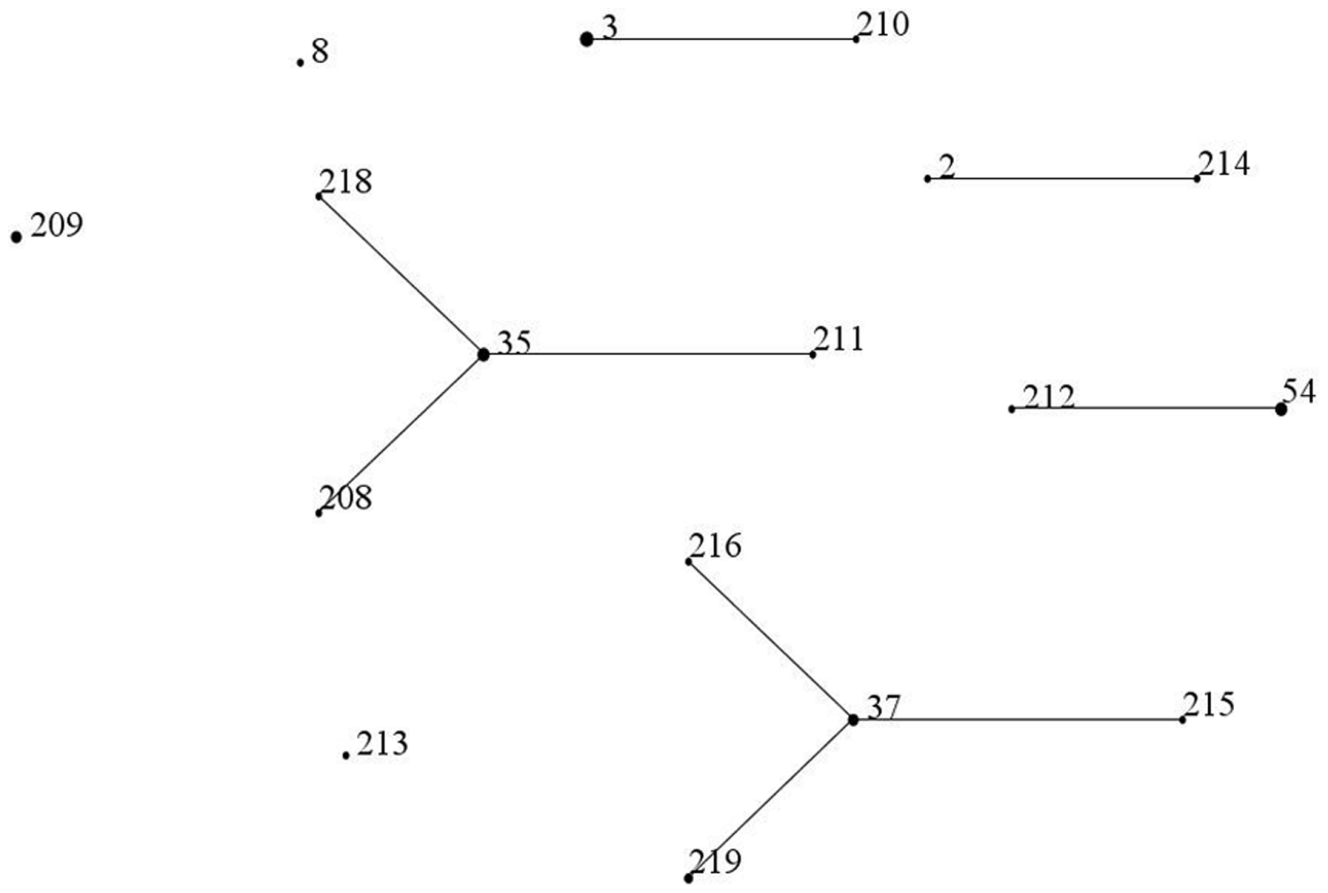
The relatedness of the STs seen in this study. The relatedness of STs were established using eBURST. ST209 and ST213 are a single-locus variant of ST5 and ST99, respectively.

A total of 17 STs, including 11 new STs, were assigned for the 28 isolates (16 STs) and the three stool DNA samples (three STs; Table 2). This appears to suggest a remarkable clonal diversity in a single unit during a relative short period. However, all of the 11 new STs were single-locus variants of known STs. most of which were also found here (Figure 1). In addition, the 17 STs identified here belonged to 4 CCs and could be clustered into 3 clades (Table 2). The diversity of toxigenic *C. difficile* in this 50-bed unit is therefore largely a reflection of a significant clonal expansion. ST3 (n = 5), ST35 (n = 4) and ST54 (n = 3) were the most common types among A+B+ strains, while A−B+ strains belonged to ST37 or its single-locus variants. This is consistent with the findings in a university hospital in Zhejiang province, China [18]. In contrast, ST35, ST37 and ST54 were rarely identified in a large European survey [4], though ST2 (ribotype 014/020) and ST3 (ribotype 001) have also been found to be prevalent. Of note, ST1 (PCR ribotype 027), a hypervirulent type widely distributed in Europe and North America, and ST11 (ribotype 078), a type associated with community-acquired CDI commonly found in Europe, were not detected in this study. In China, only one strain of ribotype 027 and one strain of ribotype of 078 have been reported up to now [20], [21].

There are several limitations of our study. First, this is a single center study, which may not represent the whole situation in China. Second, the patients were not followed after discharge from the hospital and therefore the long term outcomes and the recurrence of CDI among these cases remain unknown. Third, the number of CDI cases is limited here. Multi-center large-scale studies on CDI in ICUs are warranted.

In conclusion, to our knowledge, this is the first report of the incidence and outcome of ICU-onset CDI in China. The incidence of ICU-onset CDI in our unit is similar to those in Europe and North America in non-outbreaks settings, suggesting that CDI is likely to be a common problem in China and large-scale multi-center studies are required to reveal the burden of CDI in China. There is an urgent need to raise awareness of this disease among healthcare workers in China. Toxigenic *C. difficile* in the studied ICU belonged to a variety of STs, which is likely to represent a significant clonal expansion rather than the true clonal diversity.

## Acknowledgments

We are grateful to Wenjing Wu, Shichao Zhu, Jingwen Li, Xuelian Liao and Xiaoke Zhang for their assistance in collecting clinical data and to Tianyu Hu and Tao Chen for their technical assistance. We also thank Sally Partridge and Andrew Ginn from the Centre of Infectious Diseases and Microbiology, Westmead Hospital, The University of Sydney, Australia, for their critical reading.
